# Phytochemical and biological investigation of *Astragalus Caprinus* L

**DOI:** 10.1186/s12906-024-04484-4

**Published:** 2024-08-01

**Authors:** Walid E. Abdallah, Khaled A. Abdelshafeek, Wael M. Elsayed, Mona M. AbdelMohsen, Neven A. Salah, Heba D. Hassanein

**Affiliations:** 1 Chemistry of Medicinal Plants Department, National Research Centre, 33 El-Bohouth St., Dokki, Giza 12622 Egypt; 2 Chemistry Department, Biochemistry Division, Faculty of Science, Mansoura University, Mansoura, 35516 Egypt

**Keywords:** *Astragalus Caprinus*, Lipid fraction, Flavonoids, Antitumor, Antimicrobial activity

## Abstract

**Background:**

cultivated and wild plants are used to treat different ailments. The *Astragalus* genus is found in temperate and dry climates; thus, it is found in Egypt and the arab world. *Astragalus caprinus* has a good amount of bioactive chemicals, which may help explain its therapeutic effects in reducing the risk of consequences from disease.

**Method:**

The phytochemical investigation of the herb and roots of *Astragalus caprinus* L. included the analytical characterization for the petroleum ether components by GC/MS, unsaponifiable matter (unsap. fraction), and fatty acids (FAME) investigation by GLC analysis. Main flavonoids were chromatographically isolated from ethyl acetate and *n*-butanol extracts. In vitro antimicrobial activity has been tested against the Gram-positive bacteria *Staphylococcus aureus* and *Streptococcus mutans* for different plant extracts, the Gram-negative bacteria Pseudomonas aeruginosa and Klebsiella pneumonia, the fungus *Candida albicans* and *Aspergillus niger*, and the *Escherichia coli* bacterium. Metabolite cytotoxicity was examined using the MTT assay against HepG-2 (human liver carcinoma) and MCF-7 (breast carcinoma).

**Results:**

Identifying the important components of the herb and root petroleum ether extracts was achieved. Using column chromatography, luteolin, cosmosiin (apigenin-7-*O*-glucoside), and cynaroside (luteolin-7-*O*-glucoside) were separated and identified using UV, NMR, and Mass Spectroscopy. Root extracts displayed potential antimicrobial activity against most of the tested pathogens. Both extracts (herb and roots) were active against the MCF-7 cell line and HepG-2 cell line with IC_50_ 62.5 ± 0.64 and 72.4 ± 2.3 µg/ml, and 75.9 ± 2.5 and 96.8 ± 4.2 µg/ml, respectively.

**Conclusion:**

*Astragalus caprinus* seems to be a promising source of bioactive compounds that could potentially aid in preventing disease complications and address common health issues in developing countries. Moreover, the various parts of this plant could be utilized as natural raw materials for producing health-boosting products that could address common health issues in developing countries.

## Introduction

*Astragalus* is one of the largest genera in the plant family Fabaceae, which is widely distributed throughout the temperate and arid regions of the world [1]. *Astragalus* species growing in North Africa are Mediterranean or Arabian Saharan plants [2, 3]. *Astragalus* species have long been used in folk medicine against stomach ulcers, chronic bronchitis, hypertension, and diabetes [1]. Secondary metabolites are very important for their low cost and high biological activity [4]. Among the significant bioactive secondary metabolites found in *Astragalus* species are triterpenes, fatty acids, polysaccharides, saponins, flavonoids, and alkaloids [5–9]. Consequently, the genus possesses engaging pharmacological activities, including immunoregulatory, antitumor, antidiabetic, antioxidative, and antimicrobial, and is also considered cardioprotective [10–12].

*Astragalus caprinus* is a common species in the Fabaceae family widely distributed in northwest Africa. It extends from the northern Mediterranean region over several climatic zones, native to Egypt [13, 14]. Flavonoids were extracted and analyzed from air-dried leaves [15–17]. We examined the seeds’ lipid content, fatty acid composition, proteins, and bioactive substances [18].

In developing countries, drugs are considered to be very expensive for many people to purchase nowadays. Researchers should evaluate the endogenous plant flora of each region to obtain more data on the chemical and biological significance of indigenous plants. Egypt and the Arab world are home to the Astragalus genus, which can be found in temperate and dry climates. The presence of bioactive chemicals in *Astragalus caprinus* could explain its therapeutic effects in reducing the risk of disease consequences.

This study is designed to assign a comparative chemical composition of the herb and roots of *Astragalus caprinus* subspecies (ssp.) *langaraise* through the analytical characterization for nonpolar components as well as saponifiable and unsaponifiable compounds by GC/MS and GLC, isolation of the significant flavonoidal constituents that may correlate with its pharmacological activities. Evaluating the antimicrobial activity against selected influential bacteria and fungi and its cytotoxic activity against MCF-7 (breast carcinoma) and HepG-2 (human liver carcinoma) in vitro using MTT assay.

## Materials and methods

### Material for chromatography

Whatmann paper No. 3 MM sheets (Whatmann Ltd., Maidstone, England), Sephadex LH-20 (25–100 μm) for CC (Sigma-Aldrich Chemie GmbH, Germany), and Polyamide 30–60 mesh (Merck, Germany). Aluminum silica gel sheets G60 (with a 0.2 mm layer thickness) F254 (Fluka Chemie AG, Switzerland).

### Plant material

*Astragalus caprinus* (AC) was collected during spring and early summer (2020–2021) from El-Magtala between Mersa Matruh and Sidi Brani, Mediterranean coast, and graciously recognized by Dr. Mohammed Elgebaly, a professor at the National Research Center’s (NRC) Department of Phytochemistry and Plant Systematics. A voucher specimen was placed in the NRC Herbarium (No.209). The roots were separated from the herb and then dried in an oven at 40^o^C. After drying, the herb and roots were separately ground to a fine powder.

### Extraction and isolation of lipid constituents

The powdered herbs and roots (H and R) (500gm) were individually extracted in a soxhlet device eachusing petroleum ether (b.r. 40–60 °C). Fuller’s earth was run through the H extract to eliminate the colorful pigments. Both extracts (H and R) were filtered, dried over anhydrous sodium sulphate, and then evaporated in a vacuum at 40 °C until completely dry, yielding 1.6% and 1.1% v/w, respectively, of yellow, oily residues that were refrigerated until their GC/MS analysis. About 5 g of each oily residue was individually submitted to the saponification process to get the saponifiable and unsaponifiable fractions for the lipid content research [19]. Following saponification, the free fatty acids were methylated using the procedure outlined by Zargoun [20] to produce free acid methyl esters (FAME).

### GC/MS analysis of petroleum ether extract

The analysis of petroleum ether extract of H and R was carried out utilizing a capillary gas chromatography type Trace GC ULTRA from (Thermo Scientific) directly coupled to ISQ Single Quadruple MS and equipped with TG-5MS, nonpolar 5% phenyl methylpolysiloxane capillary column (30 m × 0.25 mm ID × 0.25 μm). The operating conditions were applied as reported by Zargoun [20].

### Gas-liquid chromatographic analysis of lipid fraction

The unsaponifiable matter of both H and R was analyzed using Agient Technologies’ 6890 N Network GC system, according to Abdelshafeek [21].

### Extraction and isolation of flavonoids

To separate the flavonoids, the defatted H marc was extracted using 80% MeOH (5 L x 3) for three days at room temperature until complete exhaustion using percolator. Filtration and concentration under reduced pressure gave the hydrophilic residue, which was dissolved in H_2_O and then partitioned successively with CH_2_Cl_2_, EtOAc, and *n*-BuOH. The different extracts were concentrated and screened using TLC. Compound 1 was obtained by chromatographing the ethyl acetate residue (5 g) on a silica gel column and then utilizing 100% methanol on a Sephadex LH-20 Column to separate the primary components. The *n*-butanol extract (6 g) was chromatographed on a polyamide column using a gradient of water/methanol (100:0 to 20:80). Fractions having similar PC profiles were pooled to afford ten fractions (F-1 to F-10). To obtain the pure isolated compounds (2–3), fractions F-5 and F-7 containing the essential compounds were repeatedly chromatographed on the Sephadex LH-20 column and eluted with various gradients of methanol/water.

### Antimicrobial assay

The antimicrobial activity of the successive extracts of both H and R were separately tested in vitro for their antibacterial activity using the agar well diffusion method against the Gram-positive bacteria, *Staphylococcus aureus* (ATCC: 13,565) and *Streptococcus mutans* (ATCC: 25,175), and the Gram-negative bacteria; *Escherichia coli* (ATCC: 10,536) and *Klebsiella pneumonia* (ATCC: 10,031) using nutrient agar medium. The antifungal activity was tested against *Candida albicans* (ATCC: 10,231) *and Aspergillus niger* (ATCC: 16,404) using Sabouraud dextrose agar medium [22]. Standard treatments for Gram-positive and Gram-negative bacteria were ampicillin and gentamicin, respectively. The standard treatment for fungal strains was the use of Nystatin. As a solvent (negative) control, DMSO was employed. The extracts were tested at a 15 mg/ml concentration against bacterial and fungal strains. This experiment was carried out in triplicate, and inhibition zones were measured in mm scale.

### MTT cytotoxicity assay

Cell cultures of HepG-2 (human liver carcinoma cell line) and MCF-7 (breast carcinoma cell line) were purchased from the American Type Culture Collection (Rockville, MD). The 3-[4,5-dimethyl-2-thiazolyl)-2,5-diphenyl-2 H-tetrazolium bromide (MTT) assay was done according to [23–25].

### Statistical analysis

Every experiment was carried out three times. GraphPad Prism® v6.0 software (GraphPad Software Inc., San Diego, CA, USA) created the concentration-response curve and fit the non-linear regression model. All results were reported as mean ± SD. IC_50_s were determined by probit analysis using SPSS software program (SPSS Inc., Chicago, IL).

## Results and discussion

### GC/MS analysis of petroleum ether extract

**Fig. 1:**
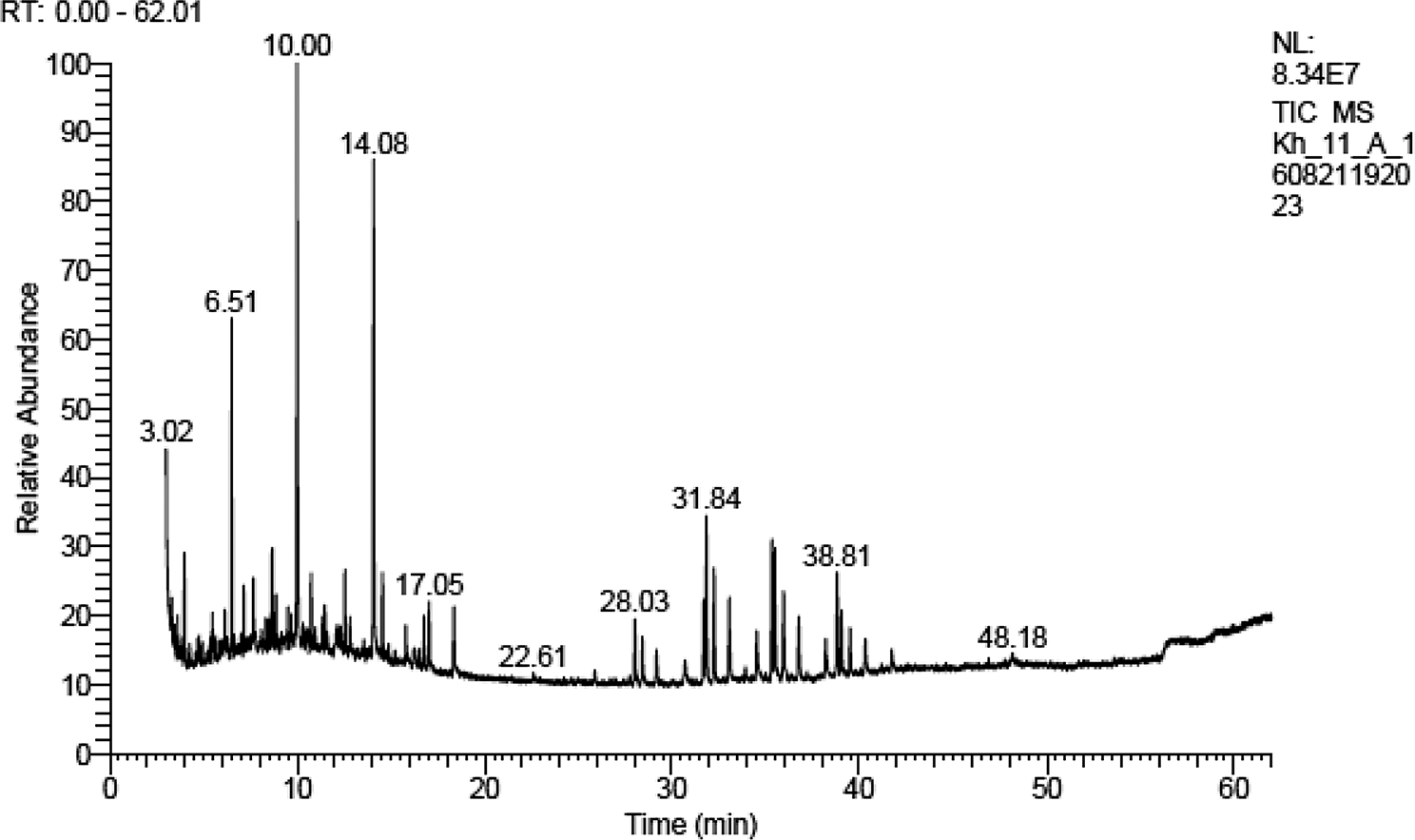
GC chromatogram of petroleum ether extract of H

**Fig. 2:**
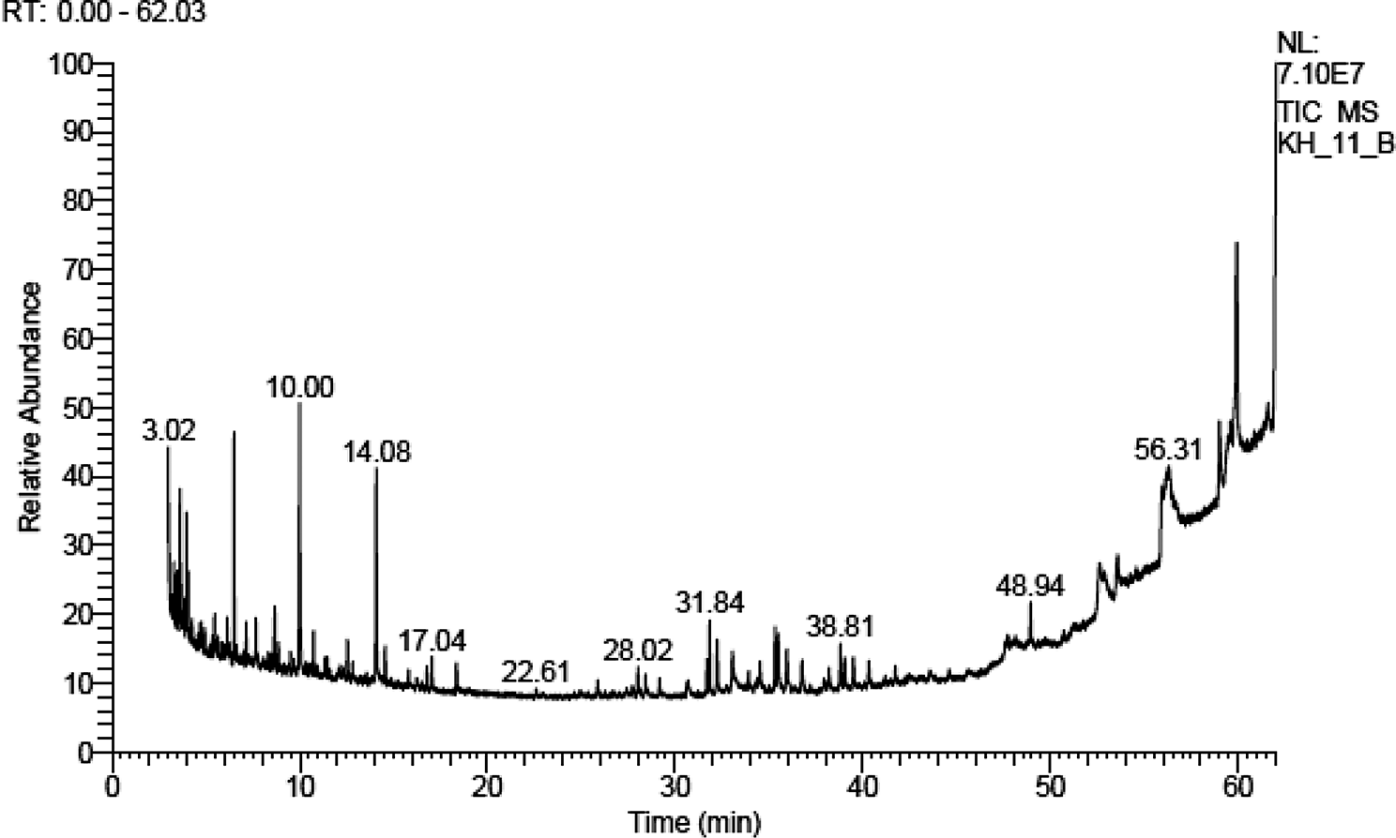
GC chromatogram of petroleum ether extract of R

**Fig. 3:**
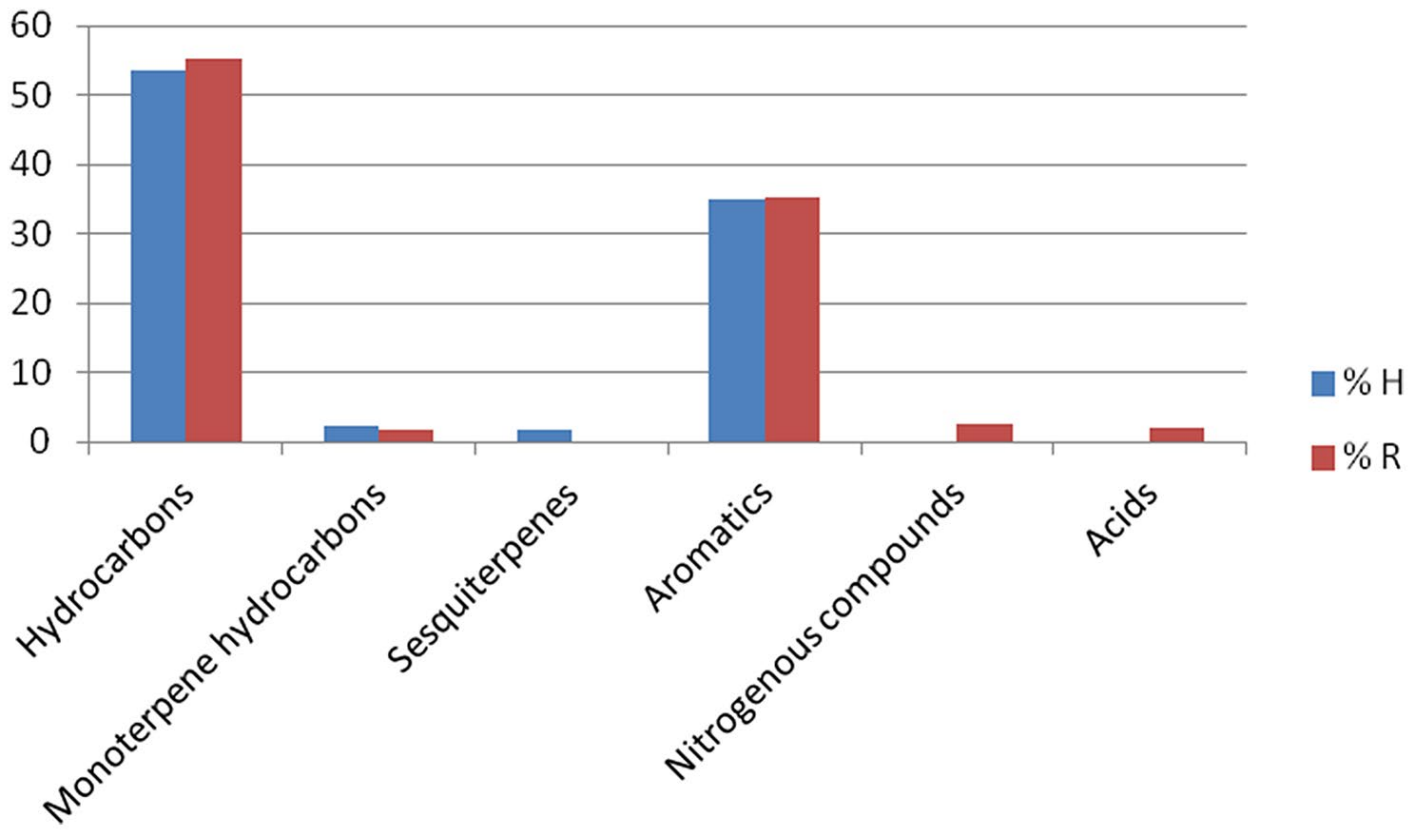
Percentage of compound classes present in both H and R

The chemical constituents of the petroleum ether extract of both H and R were identified using GC/MS analysis. Table 1 (Figs. 1 and 2) showed that the petroleum ether extracts of H and R of *A. caprinus* contained 34 and 35 compounds, respectively, belonging to different classes of phytoconstituents, including hydrocarbons which constitute 53.5% and 55.17%, respectively, with n-undecane as main one (14.32% and 11.83%, respectively), aromatic components (34.92% and 35.20% ) in which 5-phenyl undecane is the main compound (4.34% and 3,49%, respectively), only one monoterpene hydrocarbon (p- menthane) in both H and R (2.35% and 1.69%). Sesquiterpene hydrocarbons are present only in H (1.77%) and absent in R, in addition to acids, which are absent in H and present in R (2.12%). To our knowledge, these data are reported for the first time for *A. caprinus* ssp. *langarise*, Table 2; Fig. 3 shows the percentage of the different compound classes of the petroleum ether extract of H and R. Considering other species, such as *A. sieberi*, a study revealed the presence of four compounds from the pet. ether fraction with N, N-dimethyl-1-Dodecanamine (42.36%), and butylated hydroxytoluene (35.96%) as major compounds detected in the GC/MS analysis constituting 78.32% of the total peak area [26]. B-sitosterol and ceryl alcohol were isolated from the unsaponifiable fraction of *Astragalus cremophilos*, and the fatty acids were studied by GLC [27].

**Table 1:** GC/MS data of petroleum ether extract of both H and

Peak no.	*R*_t_ min	Percentage		Mass data			Compounds
		*R*	H	Chemical formula	BP.	M^+^	
1	3.89	3.05	1.54	C_9_H_20_	57	128	n-Nonane
2	4.07	2	2	C_8_H_10_	91	106	p-Xylene
3	4.28	2.48	-	C_8_H_15_NO_2_	43	157	2-ethyl-3-propyl -oxiranecarboxamide
4	5.48	1.83	2.49	C_12_H_24_	57	168	4,6,8-trimethyl − 1-Nonene
5	6.15	1.69	2.35	C_10_H_20_	56	140	P-Menthane
6	6.51	8.1	6.36	C_10_H_22_	57	142	n-Decane
7	7.15	1.43	-	C_11_H_24_	71	156	4-methyl Decane
8	7.16	-	1.24	C_10_H_22_	71	142	3,3dimethyloctane
9	7.67	1.99	2.72	C_10_H_20_	83	140	2-butyl cyclohexane
10	8.68	3.6	4.5	C_10_H_18_	67	138	*trans*-Decalin
11	8.87	1.27	1.27	C_11_H_24_	57	156	3-methyldecane
12	10	11.83	14.32	C_11_H_24_	57	156	Undecane
13	10.75	1.92	3.83	C_11_H_20_	81	152	2-methyl Decaline
14	11.48	-	1.87	C_11_H_22_	83	154	Pentyl -cyclohexane
15	12.54	1.69	-	C_12_H_26_	57	170	2-mthyl undecane
16	12.81	-	3.1	C_12_H_26_	57	170	3-methyl undecane
17	14.09	9.37	12.29	C_12_H_26_	57	170	Dodecane
18	14.56	2.96	2.17	C_13_H_28_	57	184	6-methyl dodecane
19	16.78	-	1.44	C_13_H_28_	57	184	2,3-dimethyl undecane
20	17.04	1.86	-	C_14_H_30_	57	198	4,6 dimethyldodecane
21	17.05	-	1.77	C_15_H_32_	57	212	Farnesane
22	18.36	1.45	-	C_13_H_28_	57	184	n-Tridecane
23	18.37	-	1.96	C_14_H_30_	57	198	n-Tetradecane
24	28.03	1.57	1.89	C_16_H_26_	91	218	5-phenyl decane
25	28.42	1.16	1.55	C_16_H_26_	91	218	4-phenyl decane
26	29.18	-	0.98	C_16_H_26_	91	218	3-phenyl decane
27	30.7	1.15	0.84	C_16_H_26_	91	218	2-phenyl decane
28	31.06	1.56	2.11	C_17_H_28_	91	232	6-phenyl undecane
29	31.84	3.49	4.34	C_17_H_28_	91	232	5-phenyl undecane
30	32.25	2.68	3.27	C_17_H_28_	91	232	4-phenyl undecane
31	33.06	2.03	2.44	C_17_H_28_	91	232	3-phenyl undecane
32	34.54	1.5	1.58	C_17_H_28_	91	232	2-phenyl undecane
33	35.53	3.03	3.85	C_18_H_30_	91	246	6-phenyl dodecane
34	35.96	3.48	2.64	C_18_H_30_	91	246	5-phenyl dodecane
35	36.77	2.98	1.94	C_18_H_30_	91	246	4-phenyl dodecane
36	38.12	2.76	1.24	C_18_H_30_	91	246	3-phenyl dodecane
37	38.83	-	3.12	C_18_H_30_	91	246	2-phenyl dodecane
38	39.05		1.86	C_19_H_32_	91	260	5-phenyl tridecane
39	39.5		1.38	C_19_H_32_	91	260	4-phenyl tridecane
40	40.32	2.43	-	C_15_H_13_FO_3_	91	260	3[(4Fluorophenoxy)methyl] 4-methoxybenzaldehyde
41	47.71	1.32	-	C_17_H_36_O	57	256	2-methyl 1-hexadecanol
42	48.94	2.12	-	C_18_H_32_O_2_	82	280	9,12-Octadecadienoic acid
43	52.6	2.82	-	C_21_H_44_	57	296	n-heneicosane

**Table 2:** Classes of Pet. ether ext of both H and R

No.	Classes	%	
		H	*R*
1	Hydrocarbons	53.5	55.17
2	Monoterpene hydrocarbons	2.35	1.69
3	Sesquiterpenes	1.77	-
4	Aromatics	34.92	35.2
5	Nitrogenous compounds	-	2.48
6	Acids	-	2.12

### GLC analysis of unsaponifiable fraction

**Fig. 4:**
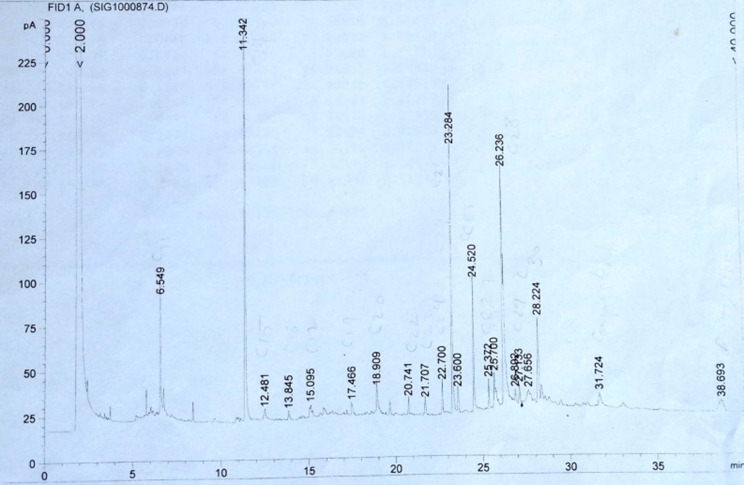
GLC chromatogram of Unsaponifiable fraction of the H

**Fig. 5:**
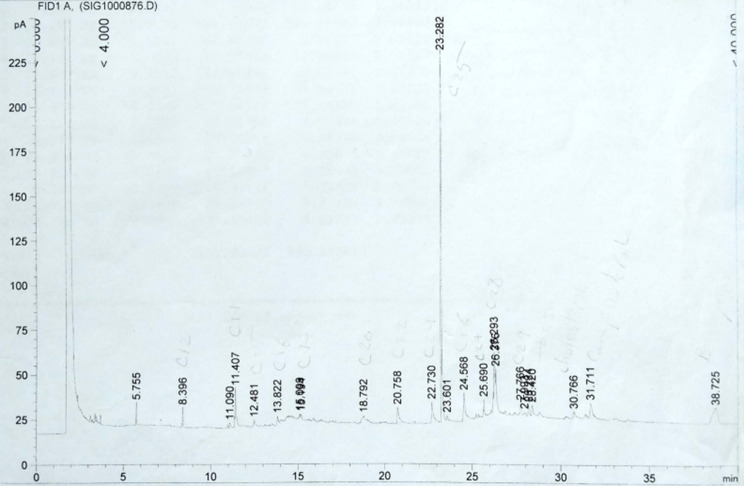
GLC chromatogram of Unsaponifiable fraction of the R

The unsaponifiable matter of both H and R was examined using GLC. The total ion chromatograms are displayed in Figs. 4 and 5 and the components are shown in Table 3. The GLC analysis of H revealed a combination of triterpenes, sterols, and hydrocarbons. The predominant hydrocarbon was C_14_ (32.98%), with the other hydrocarbons ranging from C_11_ to C_30_. Campesterol was the predominant sterol (1.62%), and only one triterpene (β- amyrin) was detected. The GLC analysis of R revealed the presence of a mixture consisting of sterols and hydrocarbons in which the predominant hydrocarbon is C_25_ (54.16%), with the other hydrocarbons ranging from C_11_ to C_30_. Campesterol was also the predominant sterol (5.67%).

**Fig. 6:**
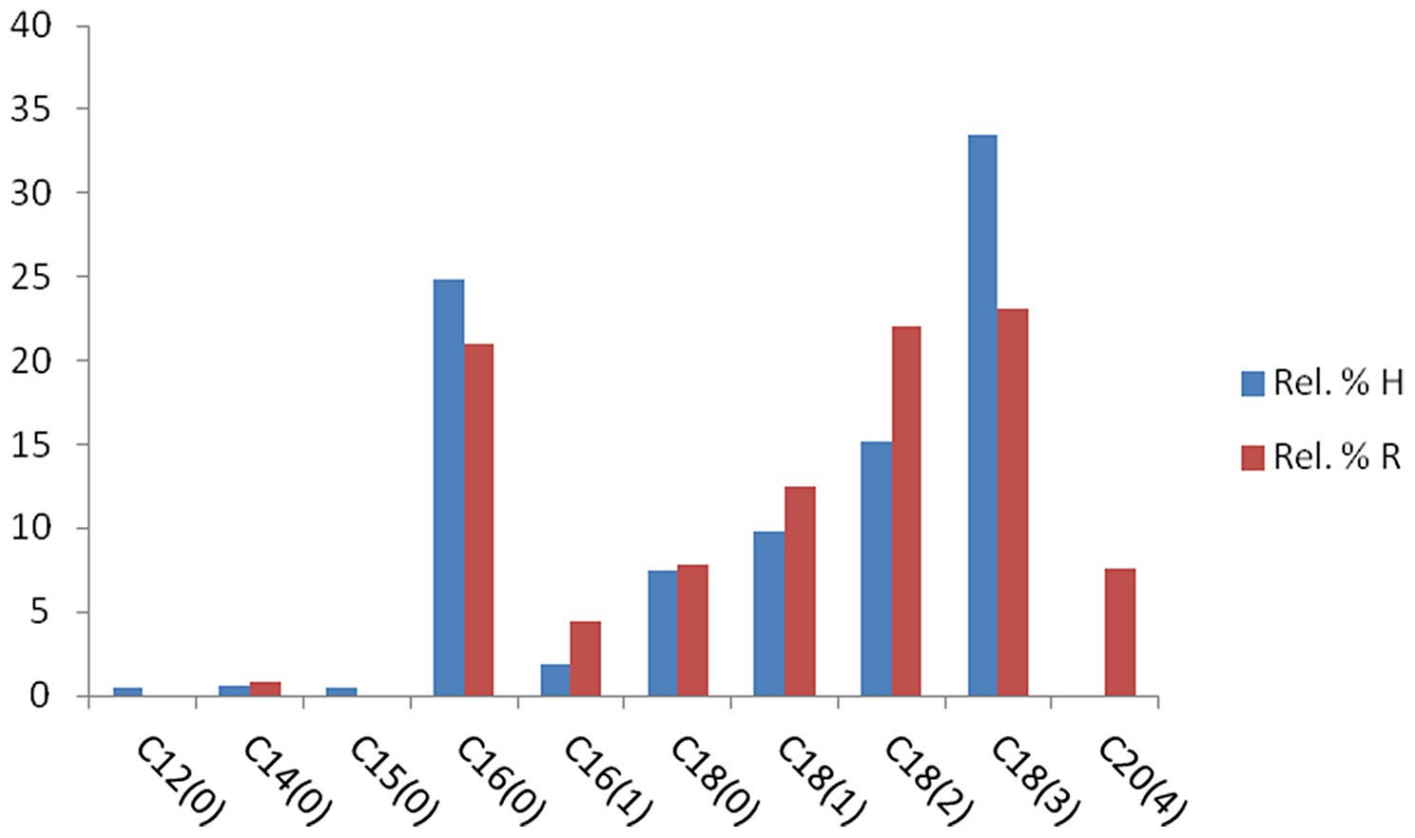
Percentage of fatty acids of both H and R

Figure 6 Displays the hydrocarbon percentage of H and R.

**Table 3:** GLC data of Unsaponifiable fractions of both H and R

Peak no.	*R*_t_ H (min.)	Relative %		Compounds H
		H	*R*	
1	8.39	7.63	-	n- Undecane (C_11_)
2	10.0	-	1.6	n- Dodecane (C_12_)
3	11.34	32.98	5.54	n- Tetradecane (C_14_)
4	12.48	1.04	1.0	n- Pentadecane (C_15_)
5	13.84	0.69	1.13	n- Hexadecane (C_16_)
6	15.09	0.81	0.63	n- Heptadecane (C_17_)
7	17.46	0.81	-	n- Nonadecane (C_19_)
8	18.90	0.23	2.90	n- Eicosane (C_20_)
9	20.74	0.92	3.02	n- Docosane (C_22_)
10	22.70	0.92	-	n- Tricosane (C_23_)
11	23.28	0.34	3.78	n- Tetracosane (C_24_)
12	24.52	13.88	54.16	n- Pentacosane (C_25_)
13	25.37	5.67	5.29	n- Hexacosane (C _26_)
14	26.23	1.15	1.89	n- Heptacosane (C_27_)
15	27.65	18.51	9.20	n- Octacosane (C_28_)
16	28.22	3.13	1.89	n-nonacosane (C_29_)
17	31.72	6.71	1.89	n-triacontane (C_30_)
18	38.69	1.62	5.67	Campasterol
19	48.18	2.89	-	β-amyrine
		99.93	99.59	

### GLC analysis of fatty acid methyl esters

**Fig. 7:**
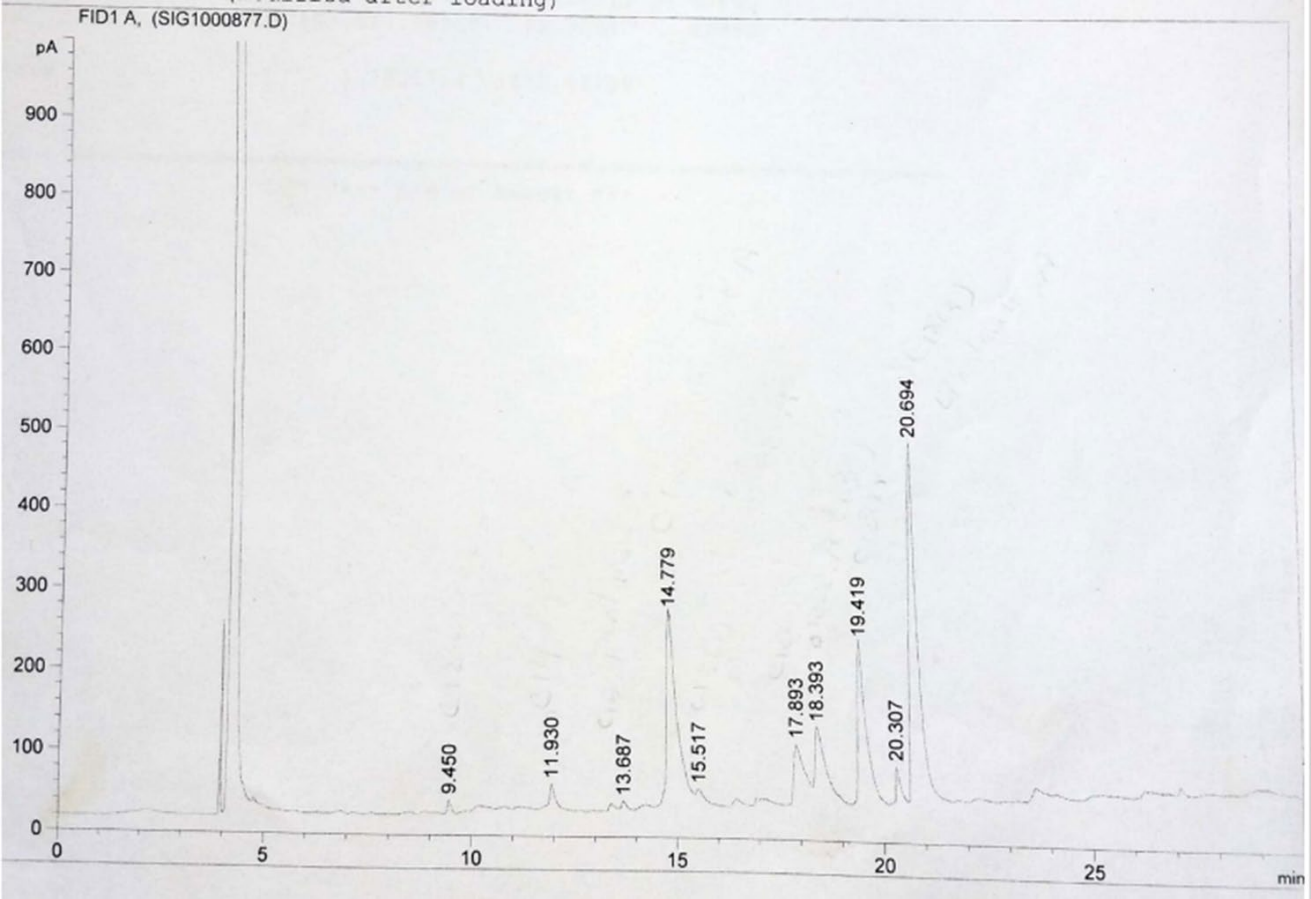
GLC chromatogram of fatty acid methyl esters of H

**Fig. 8:**
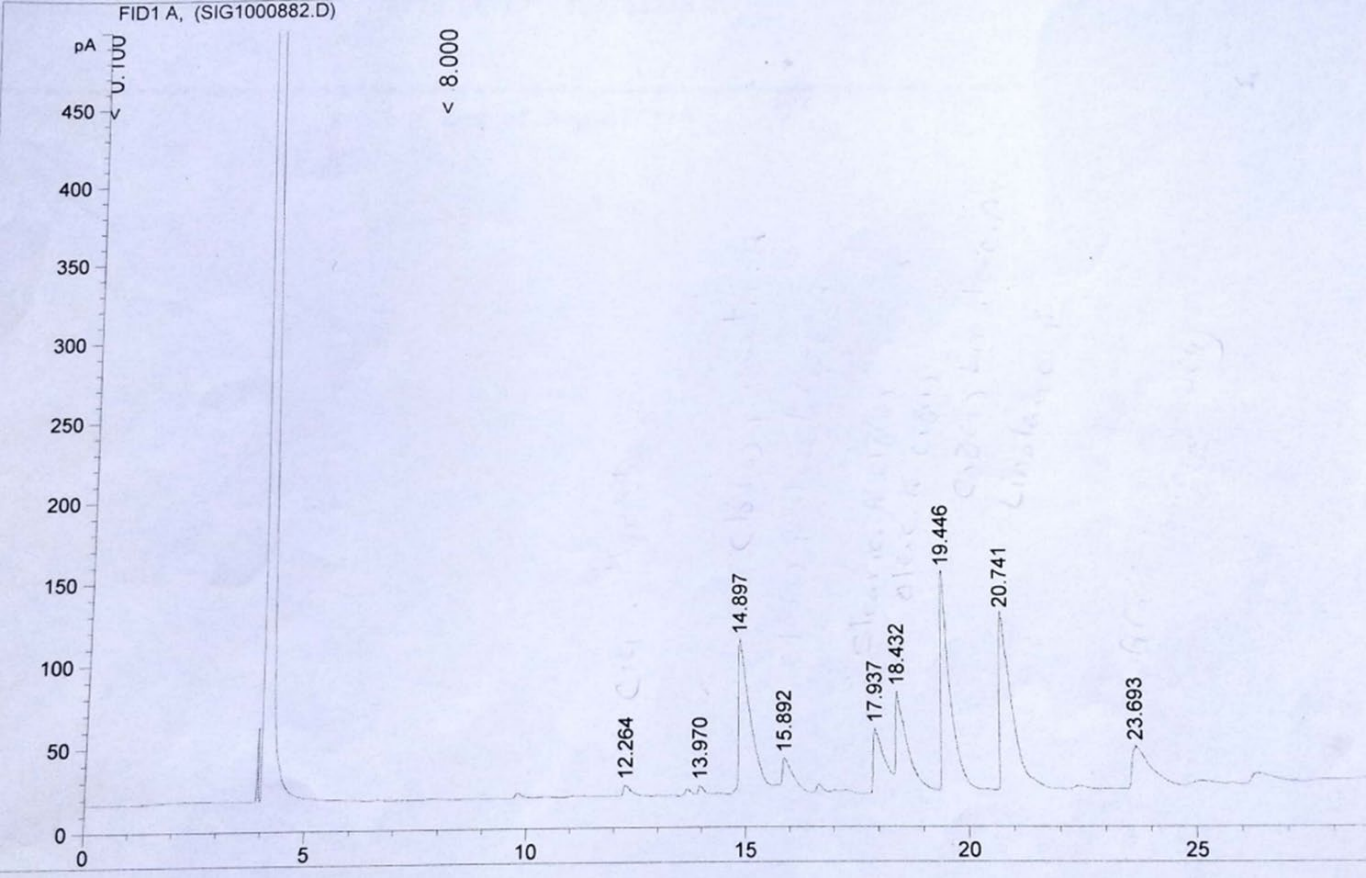
GLC chromatogram of fatty acid methyl esters of R

**Fig. 9:**
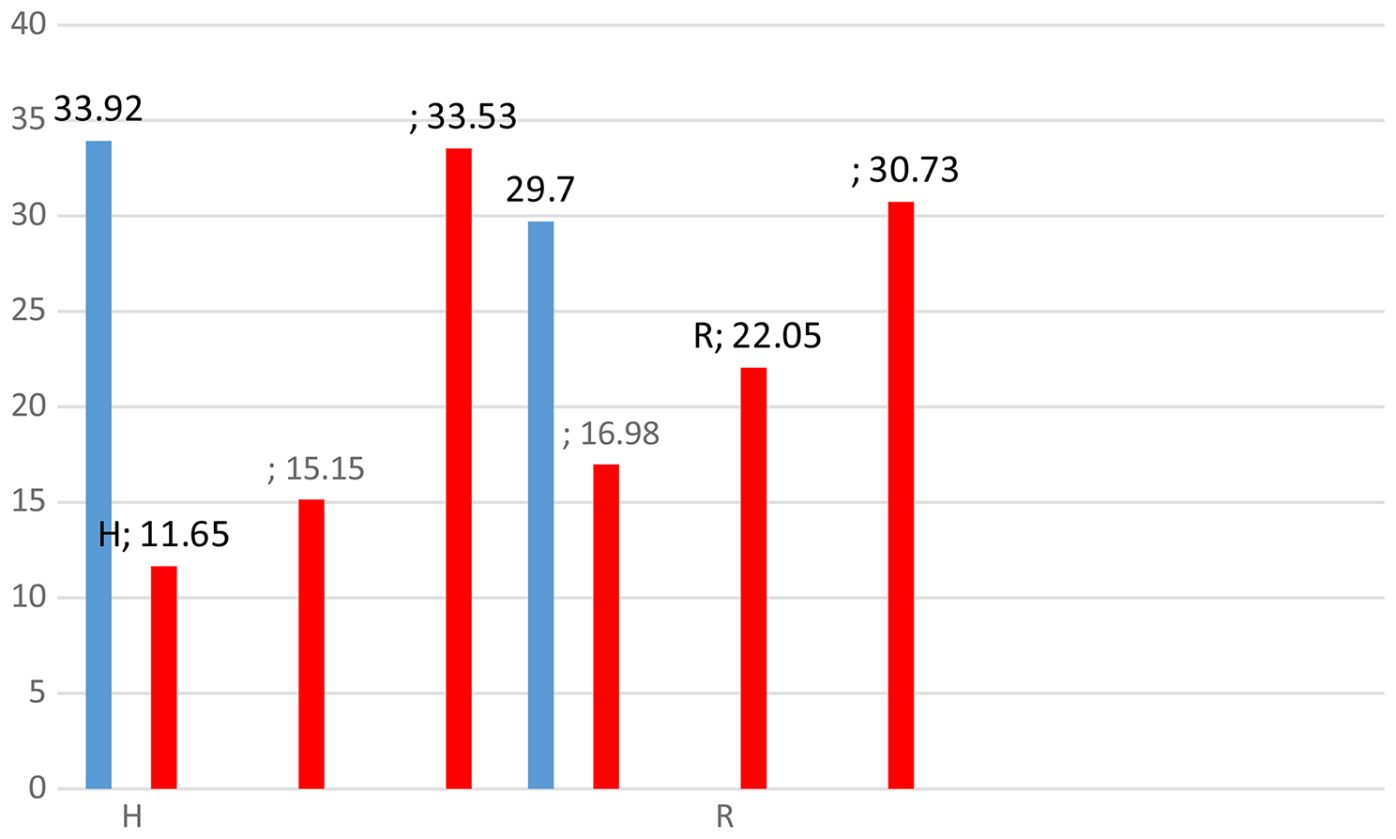
Percentage of unsaturated fatty acids in H and R

The data of the GLC analysis of the fatty acid methyl esters of both H and R (Figs. 7 and 8) displayed nine fatty acids in H, accounting for 94.25 of the total acid percentage. The primary unsaturated fatty acids are linolenic acid (33.53%) and linoleic acid (15.15%), while the major saturated fatty acid is palmitic acid (24.90%). The results of R revealed the presence of eight fatty acids, representing 99.46% of the total. Linolenic acid was the main one (23.11%), followed by Linoleic acid (22.05%). The major saturated fatty acid was palmitic acid (21.03%), as shown in Table 4. Figure 9 shows the percentage of unsaturated fatty acids in H and R.

According to Keskin and Kaçar [28], the Astragalus species have been found to contain significant amounts of palmitic (C16), linoleic (C18:2\omega-6), linolenic (C18:3\omega-3), and stearic acid (C18:0) in the roots and shoots. The fatty acid composition of *Astragalus exscapus* L. subsp. *transsilvanicus* roots were also studied by Szabo [29], and it was found that linoleic acid was the most abundant compound, followed by palmitic, oleic, and α-linolenic acids.

**Table 4:** GLC data of fatty acid methyl esters of H and R

Peak no.	*R*_t (min.)_	Rel. %						Compounds
		H			*R*			
1	9.45	0.52			-			Lauric acid C12_(0)_
2	11.93	0.55			0.83			Myristic acid C14_(0)_
3	13.68	0.52			-			Pentanoic acid C15_(0)_
4	14.77	24.90			21.03			Palmitic acid C16_(0)_
5	15.51	1.83			4.48			Palmetoleic acid C16_(1)_
6	17.89	7.43			7.84			Stearic acid C18_(0)_
7	18.39	9.82			12.50			Oleic acid C18_(1)_
8	19.41	15.15			22.05			Linoleic C18_(2)_
9	20.68	33.53			23.11			Linolenic acid C18_(3)_
10	23.69	-			7.62			Arachidonic acid C20_(4)_
Total		94.25			99.46			
Saturated fatty acid		33.92			29.7			
Unsaturated fatty acids		Mono	di	poly	mono	Di	Poly	
		11.65	15.15	33.53	16.98	22.05	30.73	
		60.33			69.76			

### Identification of flavonoids

#### Compound 1

*luteolin*, this compound was isolated as an amorphous yellowish powder, and it exhibited band-I in the UV spectrum with methanol at λ_max_ = 347 nm, which proves its flavone nature. The presence of an *ortho* dihydroxy system was confirmed through AlCl_3_/HCl spectrum, where there is a hypthochromic shift (34 nm) in the band–I relative to AlCl_3_ spectrum. The MS spectrum gave M + at m/z = 286, corresponding to the molecular formula C_15_H_10_O_6_ [30].

#### Compound 2

After re-chromatography, cosmosiin (apigenin-7-O-glucoside) was isolated as a pale yellow powder. The UV spectra of the compound in methanol and different shift reagents confirmed its flavone nature, characterized by band-I at λmax = 330nm, and the absence of an ortho dihydroxy system. The acid hydrolysis of the compound resulted in the formation of apigenin as an aglycone and glucose as a sugar. The attachment of the sugar moiety at C-7 was confirmed through band-2 at λmax = 274 nm in the sodium acetate spectrum relative to band-2 at λmax = 267 nm in methanol. The mass spectrum of the compound gave M^+^ at m/z 432, which corresponds to the molecular formula C_21_H_20_O_10_. The ^1^H-NMR data confirmed the presence of apigenin as an aglycone and only one glucose moiety as a sugar, where signals were observed at δ = 6.39 (d, J = 2.4 Hz, H-6), 6.78 (d, J = 2.4 Hz, H-8), 6.82 (s, H-3), 6.90 (d, J = 9.1 Hz, H-3’, H-5’) and 7.88 (d, J = 9.1 Hz, H-2’, H-6’). The anomeric proton of the glucose appeared at 5.39 (d, J = 6.9 Hz, H-1”) and δ C at 99.87 (C-1”). The other data obtained were consistent with previously reported data [30].

#### Compound 3

**Fig. 10:**
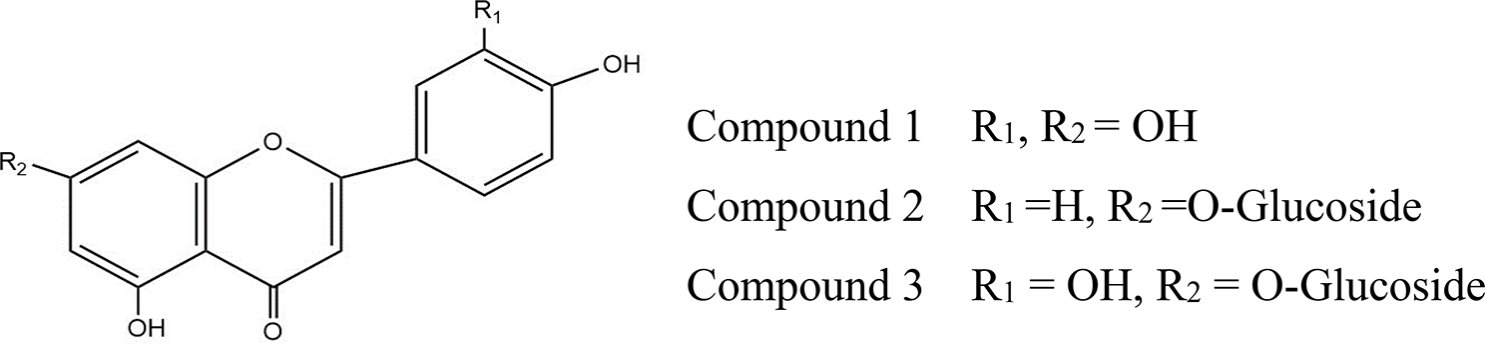
Chemical structure of isolated flavonoids

cynaroside (luteolin-7-*O*-glucoside) was isolated from the butanol fraction after column chromatography and obtained as a yellowish amorphous powder. Its chromatographic behavior and acid hydrolysis substantiate its glycosidic nature with only one glucose moiety. The UV spectra showed absorption maxima of band–I in methanol at λ_max_ = 348 nm, which proved its flavone nature; also, it established the absence of a free OH group at C-7 with no bathochromic shift in band-II (260 nm) of sodium acetate spectrum relative to methanol spectrum (256 nm). The EI-MS spectrum showed the molecular ion peak M^+^ as a small peak at m/z = 448, which fit the molecular formula C_21_H_20_O_11_ corresponding to luteolin-7-O-glucoside. The ^1^H-NMR data displayed signals at δ = 6.79 and 6.44 assigned for H-8 and H-6( J = 2.1 Hz), 6.79(s, 1H, H-3) indicates the flavone nature of the compound, 6.89 (1H, d, J = 8.2 Hz, H-5′), 7.40 (1H, d, J = 2.1 Hz, H-2′), 7.45 (1H, dd, J = 8.3, 2.1 Hz, H-6′); the anomeric proton of glucose was assigned at δ = 5.09 (1H, d, J = 7.5 Hz, H-1″) in addition to the rest of glucose protons at range of δ = 3.16–3.7, which means the presence of luteolin with glucose [30]. The chemical structure of isolated flavonoids is presented in Fig. 10.

### Antimicrobial activity

The growth absence of the microorganism (clear zone) and the diffusion of an antibiotic agent in the medium after 24 h for evaluation are related to the inhibitory zone in the well diffusion method. As indicated in Table 5, the investigation revealed that the subsequent extracts of both the H and R demonstrated differing degrees of activity against the strains that were examined, except for the Gram-negative bacteria *Klebsiella pneumonia*, the Gram-positive bacteria *Streptococcus mutans*, and the fungus *Aspergillus niger*.

### Cytotoxic assay

The in vitro cytotoxicity of *A. caprinus* 80% MeOH extracts of H and R against HepG-2 and MCF-7 human carcinoma cell lines was evaluated using an MTT assay with Doxorubicin as a positive control. The obtained results are given in Table 6.

**Table 5:** Antimicrobial activity (inhibition zone in mm) of the successive extracts of both H and R of *A. caprinus*

Microorganism	Pet. Ether		Aq.		MeOH		CHCl_3_		EtOAc		*n*-BuOH		Standard antibiotic
**Gram-negative bacteria**	H	R	H	R	H	R	H	R	H	R	H	R	Gentamicin
*Escherichia coli*	-	-	14.0	14.0	-	19.0	-	-	10.0	-	-	22.0	27 ± 0.5
*Klebsiella pneumonia*	-	-	-	-	-	-	-	-	-	-	-	-	25 ± 0.5
**Gram-positive bacteria**													Ampicillin
*Staphylococcus aureus*	-	-	-	13.0	-	21.0	-	14.0	-	-	-	21.0	22 ± 0.1
*Streptococcus mutans*	-	-	-	-	-	-	-	-	-	-	-	-	30 ± 0.5
**Fungi**													Nystatin
*Candida albicans*	-	-	18.0	10.0	17.0	-	-	-	-	-	-	19.0	21 ± 0.5
*Asperagillus Nigar*	-	-	-	-	-	-	-	-	-	-			19 ± 0.5

Pet. ether = Petroleum ether, Aq.=Aqueous, MeOH = Methanol, CHCl_3_ = Chloroform, EtOAc = ethyl acetate, *n*-BuOH = *n-*butanol

**Table 6:** The IC_50_ values of H and R methanol extracts using MTT assay

Methanol extract	MCF-7	HepG-2
	IC_50_ (µg/mL)	
H	62.5 ± 0.64	75.9 ± 2.5
R	72.4 ± 2.3	96.8 ± 4.2
Doxorubicin®	0.35	2.93

## Discussion

Many natural compounds with little or no reported adverse effects have demonstrated considerable health benefits and are found in the kingdom of plants. These therapeutic plants are abundant in phytochemical substances with critical therapeutic applications for treating various metabolic disorders. According to Tungmunnithum [31], epidemiological research has linked plant phenolics to a protective effect that lengthens average life spans and reduces the prevalence of a wide range of human disorders. Astragalus species are small shrubs or herbs with stems that develop from underground roots, either annual or perennial. These priceless plants are used as fuel, food, medicine, fodder, and decorative. Astragalus L. plants are of tremendous importance as sources of active compounds, as evidenced by the numerous studies conducted on them, including saponins, amino acids, polysaccharides, flavonoids, glycosides, organic acids, and alkaloids that have promising biological potentials [32–34]. It helps treat disorders of the urinary, respiratory, metabolic, digestive, and neurological systems and issues with the blood, circulatory system, and skin. It can be taken as a decoction, chewing, infusion, poultice, or powder [35–37].

In the present investigation, quantitation of the petroleum ether extract of H and R of *A. caprinus* was done by GC/MS. The extract contained 34 and 35 compounds, respectively, belonging to different classes of phytoconstituents, including hydrocarbons and aromatic components. Sesquiterpene hydrocarbons were present only in H and absent in R, in addition to acids, which are absent in H and present in R. The main components of the unsaponifiable fraction and the majority of the fatty acid methyl esters for both extracts were also identified. The GLC analysis of H revealed a combination of triterpenes, sterols, and hydrocarbons, while R revealed a mixture of sterols and hydrocarbons. The data of the GLC analysis of the fatty acid methyl esters of both H and R showed that the main unsaturated fatty acids were linolenic acid and linoleic acid. In contrast, the major saturated fatty acid was palmitic acid, which, following the previous study [29] and the development of novel anti-infective drugs, have represented an active research area.

According to earlier research, it may provide some health benefits because of the flavonoids it contains. The extracts of A. membranaceus roots from four different origins were examined by UHPLC-MS/MS using principal component analysis, and eighteen compounds were classified as major components, including apigenin 7-*O*-glucoside. Recently, three flavonoids were isolated and identified from the aqueous methanol extract, namely, luteolin, cosmosiin (apigenin 7-*O*-glucoside), and cynaroside, representing flavone aglycone and flavone glycosides.

The discovery of natural compounds has been recognized as a rich source of these drugs. With this, we demonstrated the antimicrobial activity of *Astragalus caprinus in* different extracts of the herb and roots. Our results revealed that the polar extracts (aqueous, methanol, and *n*-butanol) were more effective against the tested organisms, and the root extracts were considerably found to be broadly effective against the pathogens. This activity may be related to the high concentration of flavonoids and phenolic compounds in the extract, which act as antimicrobial agents through numerous mechanisms [9, 38, 39].

They provide opportunities for innovation in drug discovery because both monophenolic and polyphenolic compounds from various plants have been demonstrated to inhibit, block, or attenuate the initiation, progression, and spread of cancer in normal or pre-neoplastic tissues. They have also been shown to interfere with different stages of carcinogenesis in vitro and in vivo studies through a process known as radical scavenging.

It’s interesting to note that the scientific community has been closely studying *Astragalus* species due to their potential for cytotoxicity. Recent research has shown that the genus possesses intense anticancer activity, which is relevant to its cytotoxic potential [40]. One particular study found that *Astragalus* plants exhibited cytotoxic ability against MCF-7 and HepG-2 cancer cell lines, indicating the function of its phenolic chemicals [12, 41]. It’s worth noting that more research is necessary to search for new anticancer drugs. This present scientific study has gathered data that shows *Astragalus caprinus* is high in bioactive polyphenols that may be responsible for various pharmacological activities. As a result, it needs to be further investigated for its potential uses as a natural health-promoting agent.

## Conclusion

*Astragalus caprinus* seems to be a promising source of bioactive compounds that could potentially aid in preventing disease complications. Moreover, the various parts of this plant could be utilized as natural raw materials for producing health-boosting products that could address common health issues in developing countries.

Further toxicity investigation is recommended for this plant to be one of the promising plants in the pharmacological industry.

## Data Availability

All data generated or analyzed during this study are included in this published article.

## Declarations

### Author details

1-Chemistry of Medicinal Plants Department, National Research Centre, 33 El-Bohouth St., Dokki, Giza 12622, Egypt. 2-Al-Baha University, Faculty of Sciences and Arts, Almukhwah, Al-Baha, Saudi Arabia. 3-Chemistry Department, Biochemistry Division, Faculty of Science, Mansoura University, Mansoura, 35516, Egypt.
