# Correction: Phytochemical and biological investigation of *Astragalus Caprinus* L

**DOI:** 10.1186/s12906-024-04625-9

**Published:** 2024-08-22

**Authors:** Walid E. Abdallah, Khaled A. Abdelshafeek, Wael M. Elsayed, Mona M. AbdelMohsen, Neven A. Salah, Heba D. Hassanein

**Affiliations:** 1 Chemistry of Medicinal Plants Department, National Research Centre, 33 El-Bohouth St., Dokki, 12622 Giza Egypt; 2 Chemistry Department, Biochemistry Division, Faculty of Science, Mansoura University, Mansoura, 35516 Egypt

**Correction: BMC Complement Med Ther 24, 294 (2024)**

10.1186/s12906-024-04484-4

Following publication of the original article [1], the authors reported an error in Funding section.

The said section should be removed as the study does not received any funding support.

The original article has been corrected.
